# An Aligned Patterned Biomimetic Elastic Membrane Has a Potential as Vascular Tissue Engineering Material

**DOI:** 10.3389/fbioe.2020.00704

**Published:** 2020-06-30

**Authors:** Juanjuan Tan, Jing Bai, Zhiqiang Yan

**Affiliations:** ^1^School of Chemistry and Chemical Engineering, State Key Laboratory of Metal Matrix Composite Materials and Shanghai Key Lab of Electrical Insulation and Thermal Ageing, Shanghai Jiao Tong University, Shanghai, China; ^2^Joint Research Center for Precision Medicine, Shanghai Jiao Tong University Affiliated Sixth People’s Hospital South Campus, Shanghai, China; ^3^Central Laboratory, Southern Medical University affiliated Fengxian Hospital, Shanghai, China

**Keywords:** vascular tissue engineering, nanoparticles, anthracene-grafted SBS, HUVECs, biocompatibility

## Abstract

Cardiovascular disease is the leading cause of death worldwide, with an annual mortality incidence predicted to rise to 23.3 million worldwide by 2030. Synthetic vascular grafts as an alternative to autologous vessels have shown satisfactory long-term results for replacement of large- and medium-diameter arteries, but have poor patency rates when applied to small-diameter vessels. Nanoparticles with low toxicity, contrasting agent properties, tailorable characteristics, targeted/stimuli- response delivery potential, and precise control over behavior (via external stimuli such as magnetic fields) have made possible their use for improving engineered tissues. Poly (styrene-block-butadiene-block-styrene) (SBS) is a kind of widely used thermoplastic elastomer with good mechanical properties and biocompatibility. Here, we synthesized anthracene-grafted SBS (SBS-An) by the method for the fabrication of a biomimetic elastic membrane with a switchable Janus structure, and formed the patterns on the surface of SBS-An under ultraviolet (UV) light irradiation. By irradiating the SBS-An film at different times (0, 10, 20, 30, 60, and 120 s), we obtained six well-ordered surface-patterned biomimetic elastic film with SBS-An at different heights in the thickness direction and the same distances of intervals (named sample-0, 10, 20, 30, 60, and 120 s). The structural effects of the SBS-An films on the adhesion and proliferation of human umbilical vein endothelial cells (HUVECs) were studied, and the possible mechanism was explored. When the HUVECs were cultured on the SBS-An films at different heights in the thickness direction, the sample-30 s with approximately 4 μm height significantly promoted adhesion of the HUVECs at the early stage and proliferation during the culture period compared with the samples of the lower (0, 10, and 20 s) and higher (60 and 120 s) heights. Consistent with this, the sample 30 s showed a higher stimulatory effect on the proliferation- and angiogenesis-related genes. These results suggest that SBS-An with appropriate height could efficiently control bioactivities of the biomimetic elastic membrane and might have great potential in vascular tissue engineering application.

## Introduction

Cardiovascular diseases are a serious threat to human health. The number of deaths caused by cardiovascular disease is as high as 15 million worldwide every year, ranking first among various causes of death. Another statistic enumerates approximately 850,000 vascular reconstruction operations in the world each year, most of which use autologous blood vessels, though postoperative data show that the surgical effect is not ideal given the scarcity of autologous blood vessel sources (McBane et al., 2012; Schwann et al., 2012). Therefore, the research and development of artificial blood vessels have great application prospects and social significance.

An artificial blood vessel is a substitute that can be used to replace or repair a diseased blood vessel, and its source does not belong to the tissue or organ contributed by the host’s own or a foreign body. Thus, the design of the artificial blood vessel should meet certain performance requirements, such as a suitable microporous structure on the surface and the long-term compliance of the blood vessel in the body (Chen et al., 2001; Nerem, 2004). Therefore, materials play a vital role in the successful application of tissue engineering, and choosing the right materials will have a profound impact on the regeneration of new tissues in the human body. The main challenge for tissue engineering is to develop materials that can promote the required cells and identify tissue behavior (O’Brien, 2011). The development of artificial blood vessels began in the early 20th century. Scholars from various countries first used tubular materials made of metal, glass, polyethylene, silicone rubber, and other materials for a large number of animal experiments (Nerem and Seliktar, 2001). However, they were not widely used in clinical practice because of their susceptibility to intraluminal thrombosis in the short term. In 1952, Voorhees et al. (1952) first studied the use of vinylon as a vascular prosthesis, which changed the impermeability of the previous vascular wall. In the next few years, Voorhees et al. did a large number of clinical trials to develop a meshed artificial blood vessel, which is a milestone in the history of the development of vascular substitutes. Subsequently, experts tested many materials such as PVC (polyvinyl chloride), polyacrylonitrile (acrylic), silk, nylon, and viscose (Guidoin, 1992). Artificial blood vessels made of polyacrylonitrile (acrylic) and nylon will degrade in the body; thus, these two materials were quickly eliminated. In addition, polyurethane (PU) has been used in tissue engineering as a vascular implant for 30 years due to its excellent blood compatibility, mechanical strength, and ideal long-term patency. It is considered by many researchers as an ideal artificial blood vessel material. However, some studies have pointed out that after long-term implantation, aging degradation and calcification can occur, which results in material cracking (Kannan et al., 2005). Although the clinical demand for bioengineered blood vessels continues to rise, currently there are still limited choices for blood vessels. Therefore, finding new materials remains the focus of research.

Natural blood vessels are mainly composed of three layers: intima, media, and adventitia (Li et al., 2014). The innermost layer is the endothelial cell layer. Intact endothelial cells adhere to the basement membrane containing collagen and laminin to prevent infection and thrombosis. Vascular endothelial cells are not only a physical barrier on the surface of blood vessels, but more importantly, they play an indispensable function and role in the regulation of vasoconstriction and the maintenance of the balance of the coagulation-fibrinolytic system. Constructing an endothelial cell layer with normal morphology and physiological functions is an important means to solve the thrombosis after the in vivo implantation of tissue-engineered blood vessels. The endothelialization of tissue-engineered blood vessels can effectively resist thrombosis and inhibit intimal hyperplasia (Ballyk et al., 1997; Chlupác et al., 2009). Therefore, a hot spot in the study of vascular stent biocompatibility is the rapid endothelialization of the stent surface and the maintenance of normal endothelial function after endothelialization.

In recent years, micrographics technology has received increasingly extensive attention, particularly in the fields of microelectronics, optics, tissue engineering, and biochip manufacturing due to its important research value. The topological surface structure of biological materials, such as their surface roughness, pore size, pore distribution, shape, size, and micro-pattern orientation greatly influence cell adhesion, proliferation, and differentiation (Lim and Donahue, 2007; Nel et al., 2009). Experiments have shown that a stripe structure with a large pitch (100 μm) can promote endothelial cell orientation, but does not form a capillary lumen structure. Among them, smaller-spaced stripe structures (10–50 μm) are more oriented to induce the growth of endothelial cells, and can promote the formation of a tubular structure of the lumen and a vascular network (Lei et al., 2013). Whited et al. studied the effect of PCL/gelatin electrospun materials with different fiber diameters and orientations on the angiogenic capacity of surface-grown ECs (Whited and Rylander, 2014). Their immunofluorescence staining image results showed that EC on the single-layer growth on the material surface, and the state of cell alignment and stretching increased with the degree of fiber orientation. Along the arrangement direction of the fibers, thicker actin bundles (F-actin bundles) were aligned, and strong expression of cadherin (VE-cadherin) was also observed at the junction between cells. The results indicated that the electrospun fiber materials with a directional arrangement structure helped enhance the adhesion ability of the ECs (Whited and Rylander, 2014). The aforementioned studies have demonstrated that the micro-pattern structure on the surface of the material effectively influenced and further regulated the cell-growth behavior on the material.

Interestingly, a series of studies have demonstrated that poly SBS is a widely used thermoplastic elastomer with good mechanical properties and biocompatibility, and more importantly, lower biotoxicity (Cho and Paul, 2001; Fu and Qutubuddin, 2001; Kim et al., 2001; Novak and Florián, 2004). However, in our previous study, a kind of biomimetic Janus membrane was fabricated with anthracene-grafted SBS and carbon nanotubes (CNTs), and the gradient Janus structure was obtained via the combination of UV light-induced dimerization of anthracene and control of the UV light penetration depth by the CNTs (Bai and Shi, 2017). These results demonstrated that this Janus membrane could be used as a shape actuator due to the Janus structure-induced impetus in its thickness direction. By designing different geometrical shapes and UV light irradiation conditions (time and location) or stimulated regions, the Janus membrane achieved more complex and diversified shape shifting or morphing triggered by the solvent effect or shape memory mechanism. In addition, we developed a new approach for making two-/three-dimensional (2D/3D) latent photopatterned morphologies on the modified SBS films. Therefore, we intend to investigate the potential of the SBS-An films as tissue-engineered vascular material. In this study, the branching ratio of anthracene was fixed at 10% of the double bonds on SBS chains, which can be crosslinked under UV light irradiation to easily form the pattern on the surface. Finally, the structure effects of the SBS-An films on the adhesion and proliferation of HUVECs were studied, and the possible mechanism was explored.

## Materials and Methods

### Materials

Toluene, 4-methylbenzenesulfonic acid, 4-dimethylaminopyridine, pyridine, and dichloromethane were purchased from Sinopharm Chemical Reagent Co., Ltd (Beijing, China). SBS (Mw∼153 000–185 000, 70 wt % PB block), 2-Mercaptoethanol, and succinic anhydride were purchased from Sigma-Aldrich. 9-anthracenemethanol was purchased from Alfa Aesar. All the reagents were used as received.

### Preparation of the Anthracene-Grafted SBS

The UV light sensitive polymer (SBS-An) was prepared as our previous work (Bai and Shi, 2017). First, the hydroxyl-branched SBS was synthesized via the thiol-ene click reaction. The anthracene group was then grafted to the polymer chains via esterification. In the manuscript, the branching ratio of anthracene was fixed at 10% of the double bonds on the SBS chains. Due to the dimerization of anthracene, this kind of polymer can be crosslinked under UV light irradiation, while was utilized to form the pattern on the surface.

### Preparation of the Surface Patterns

**FIGURE 1:**
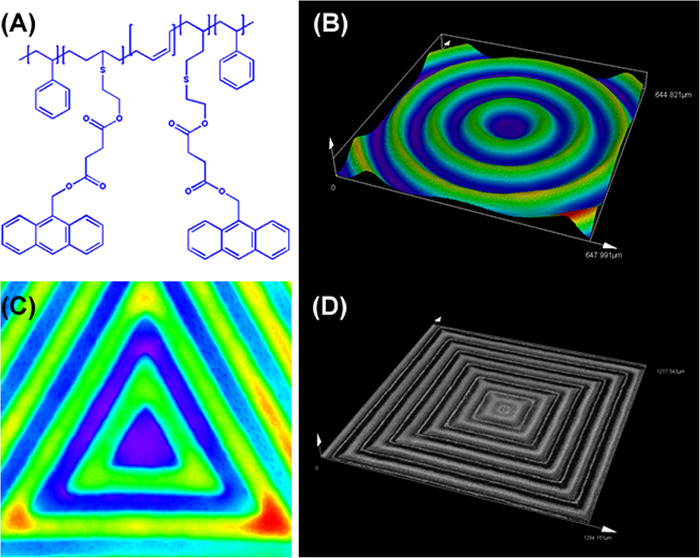
**(A)** The chemical structure of the anthracene modified SBS. Laser scanning confocal microscopy (LSCM) images showing patterns written with different geometric configuration controlled with masks. **(B)** Positive and negative hybrid concentric rings (size width/space: 50/50 μm). **(C)** Positive and negative hybrid concentric triangles (size width/space: 100/100 μm). **(D)** Positive and negative hybrid concentric quadrangles (size width/space: 50/50 μm). The thickness of the polymer blend film was ≈200 μm. The intensity and exposure time of 365 nm UV light were ≈50 mW/cm^2^ and 30 s, respectively.

According to our previous study (Bai et al., 2019), the patterns can be written on the surface as shown in Figure 1. In detail, The SBS-An was dissolved in toluene, and the solution was cast on the glass plate. The films were then heated at approximately 80°C for 12 h to evaporate solvent. To write the patterns, the films were exposed to 365 nm UV light covered with a photomask at different times (0, 10, 20, 30, 60, and 120 s; marked as sample-0, 10, 20, 30, 60, and 120 s) to initiate the photo-dimerization of An to obtain a well-ordered surface pattern that transferred the pattern on the mask with different height in the thickness direction without cumbersome processing steps. The pattern on the surface of films can be obtained almost the same as the designed masks.

### Cell Culture

According to a previous description with modification (Tan et al., 2017), the HUVECs were isolated from the human umbilical cord vein with 0.1% type I collagenase. The HUVECs were cultured in the endothelial cell medium (ECM) containing 5% FBS and 1% endothelial cell growth supplement kit (Sciencell, Carlsbad, CA, United States) at 37°C with 5% CO_2_. The purity of the ECs was confirmed using the von Willebrand factor (vWF) antibody (Abcam, Cambridge, United Kingdom). Cells from passages 3–5 were employed for all experiments.

### HUVEC Adhesion Assay

To evaluate the influence of the aligned patterned biomimetic elastic membrane on cell adhesion, samples were first sectioned into 10 × 10 mm^2^ squares, and then soaked in ethanol for 0.5 h. The samples were then soaked in 75 vol% medical alcohol solution for another 2 h for sterilization and washed out with a large amount of sterile water prior to seeding cells on the samples.

The cell adhesion assay was performed as previously described (Winterbone et al., 2009). Briefly, the HUVECs were plated into the surfaces of those samples at a density of 2 × 10^4^ cells per well in a 24-well culture plate. Following incubation for 6 h at 37°C in 5% CO_2_, the wells were washed three times with 0.2 mL PBS, stained with 0.1% crystals, and the number of adherent cells in four high power fields of view were observed using a Leica BX-61 fluorescence microscope at ×100 magnification (Leica, Germany).

### HUVEC Proliferation Assay

To investigate the influence of the aligned patterned biomimetic elastic membrane on cell proliferation, the samples were treated as described above. HUVECs were trypsinized and plated onto the surfaces of the samples at a density of 2 × 10^4^ cells per well in a 24-well culture plate, and incubated in an atmosphere consisting of 5% CO_2_ in air at 37°C for 72 h. The absorbance of HUVECs was determined with a CCK8 assay (Dojindo Molecular Technologies, Japan) according to the instructions of the manufacturer, and measured at 450 nm using an enzyme-linked immunosorbent assay plate reader (Bio-TEK, United States). Cells cultured in normal condition were regarded as controls.

### Quantitative Real-Time Polymerase Chain Reaction (qRT-PCR)

To investigate the effect of aligned pattern of biomimetic elastic membrane on the proliferation and differentiation of HUVECs, the proliferation-related genes (p21, proliferating cell nuclear antigen (PCNA), cyclin-dependent kinase 2(CDK2), and Cyclin A2) and the angiogenesis-related genes [vascular endothelial growth factor receptor 2 (KDR), endothelial nitric oxide synthase (eNOS), and vascular endothelial cadherin (VE-cad)] from the cells cultured on different samples for 72 h were detected by qRT-PCR. TRIzol Reagent (Invitrogen, United States) was used to extract total RNAs according to the instructions of manufacturer. The concentration and purity of RNA were measured by a nanodrop 1000 reader (Thermo Scientific, United States). qRT-PCR was performed with the 2 × SYBR green master mix (Takara, Japan) with a 7500 Real-Time PCR System (Applied Biosystems, United States). After an initial incubation step of denaturation for 1 min at 95°C, 40 cycles (95°C for 15s, 60°C for 30s, 72°C for 20 s) of PCR were performed. Reactions were performed in triplicate. The PCR primer sequences of the above genes and glyceraldehyde 3-phosphate dehydrogenase (GAPDH) are summarized in Table 1. All fold changes were calculated by the method of 2^–ΔΔCt^. GAPDH was used as a housekeeping gene. Data were normalized to GAPDH mRNA expression of each condition and were quantified relative to the corresponding gene expression from the control samples (cells cultured on the surface of sample-0 s or in normal condition).

**TABLE 1:** Primer sequences used in qRT-PCR.

**Gene**	**Gene bank**	**Primer sequences**	***T*_m_ (°C)**
eNOS	NM_001160111	F: 5′-TGTCCAACATG CTGCTGGAAATTG-3′	55
		R: 5′-AGGAGGTCTTCTTCCT GGTGATGCC-3′	
KDR	NM_002253	F: 5′-GTGATCGGAAA TGACACTGGAG-3′	60
		R: 5′-CATGTTGGTCACTA ACAGAAGCA-3′	
VE-Cadherin	NM_001795	F: 5′-GGCTCAGAC ATCCACATAACC-3′	63
		R: 5′-CTTACCAGGGCGT TCAGGGAC-3′	
Cyclin A2	NM_001237	F: 5′-CGCTGGCG GTACTGAAGTC-3′	60
		R: 5′-GAGGAACGGTGACA TGCTCAT-3′	
P21	NM_000389	F: 5′-TGTCCGTCAGAACCC ATGC-3′	63
		R: 5′-AAAGTCGAAGTTCC ATCGCTC-3′	
PCNA	NM_002592	F: 5′-CCTGCTGGGA TATTAGCTCCA-3′	60
		R: 5′-CAGCGGTAGGTG TCGAAGC-3′	
CDK2	NM_001290230	F: 5′-CCAGGAGTTACT TCTATGCCTGA-3′	58
		R: 5′-R TTCATCCAGGGGA GGTACAAC-3′	
GAPDH	NM_002046	F: 5′-ACAAGATGGTGAA GGTCGGTGTGA-3′	60
		R: 5′-AGCTTCCCATTCTC AGCCTTGACT-3′	

### Statistical Analysis

Experimental data were expressed as a means ± standard deviation (SD). Three independent experiments were performed, and at least three samples per each test were taken for statistical analysis. The statistical significance was calculated by a Student’s *t*-test or one-way ANOVA using GraphPad Prism 6.0 (GraphPad Software, San Diego, CA, United States). Differences were considered significant when *p* < 0.05 (^∗^), *p* < 0.01 (^∗∗^).

## Results

### The Preparation of the Patterns on the Polymer Films

**FIGURE 2:**
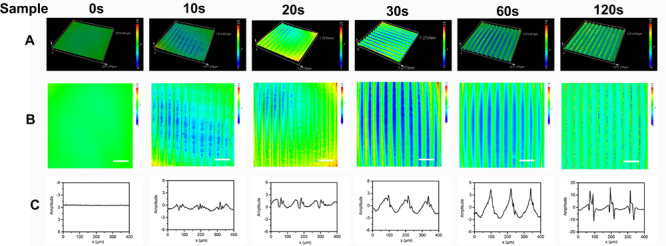
**(A)** 3D images of sample-0, 10, 20 30, 60, and 120 s by LSCM, respectively. **(B)** 2D images of sample sample-0, 10, 20 30, 60, and 120 s by LSCM, respectively. **(C)** Height of the sample-0, 10, 20 30, 60, and 120 s, respectively. Scale bar: 100 μm.

As shown in Figure 1, Figure 1A shows the chemical structure of the anthracene modified SBS. SBS-An films can be patterned with different geometric configuration controlled with masks, such as rings (Figure 1B), triangles (Figure 1C), and hexagons (Figure 1D). Due to the different irradiating time, the patterns exhibited different height which increased with the irradiation time as reported in our previous work. Figure 2 shows the 3D/2D images and the height of SBS-An films exposed to 365 nm UV light covered with a quadrangular photomask for 0, 10, 20, 30, 60, and 120 s. With the same distance of interval(50 μm), the height of samples 0-, 10-, 20-, 30-, 60-, and 120-s were 0, 1.36 ± 0.12, 2.56 ± 0.09, 4.47 ± 0.08, 7.22 ± 0.05, and 22.86 ± 0.28 μm, respectively.

### Effect of the Aligned Patterned Biomimetic Elastic Membrane on Cell Adhesion

**FIGURE 3:**
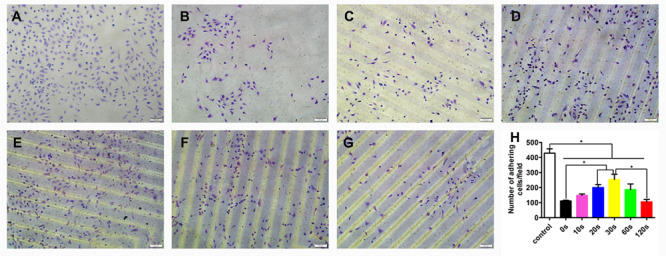
Adhesion of HUVECs on the biomimetic elastic membrane with different height in the thickness direction (*p < 0.05). **(A–G)** HUVECs were stained with 0.1% crystals after cultured in normal condition and on the sample-0, 10, 20, 30, 60, and 120 s for 6 h. Scale bar: 100 μm. **(H)** Quantitation of adhesive cells. The data are shown as the mean ± SD of the number of adhesive cells from three independent experiments. *p < 0.05 represents statistical significance.

The effect of the aligned patterned biomimetic elastic membranes on the adhesion of HUVECs were evaluated, as shown in Figure 3. On the whole, the adhesion of HUVECs cultured on the SBS-An films were lower than those in the normal condition. The number of adhesive HUVECs cultured on the sample-20 and 30 s biomimetic elastic membranes were higher than those on the sample-0 and 10 s biomimetic elastic membrane. Specifically, the number of adhesive HUVECs on sample-30 s biomimetic elastic membrane was a 2.28-fold increase compared with those on the sample-0 s. By contrast, the adhesion of HUVECs cultured on the sample-10 and 120 s biomimetic elastic membranes were no significant difference compared with those on the sample-0 s.

### Effect of the Aligned Patterned Biomimetic Elastic Membrane on Cell Proliferation

**FIGURE 4:**
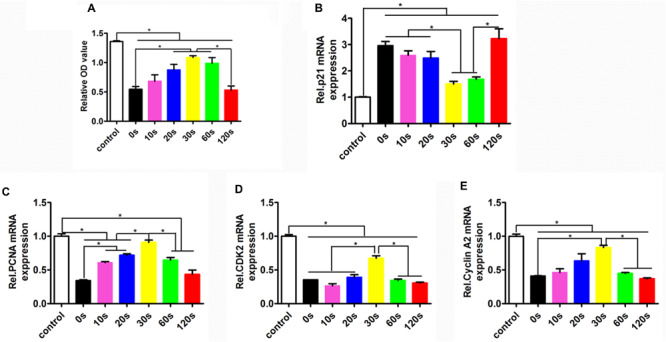
Proliferation of HUVECs on the biomimetic elastic membrane with different height in the thickness direction. **(A)** The proliferation of HUVECs were assayed by CCK8 after cultured in normal condition and on the sample-0, 10, 20, 30, 60, and 120 s for 72 h. The mRNA expression of proliferation-related genes in HUVECs shown in each panel were **(B)** p21, **(C)** PCNA, **(D)** CDK2, and **(E)** cyclin A2, respectively (*p < 0.05).

To determine the HUVEC proliferation on the biomimetic elastic membrane, the viabilities of HUVECs were determined by CCK8 assay. Figure 4 summarized the cell viability after incubation for 72 h. Overall, the proliferation of HUVECs cultured on the SBS-An films were lower than those in normal condition. However, after 72 h, the number of HUVECs cultured on sample-20, 30, and 60 s were significantly higher than those cultured on the sample-0 and 120 s, which indicates that those samples have stimulatory effects on HUVEC proliferation, while the number of HUVECs cultured on sample-10 and 120 s had no significantly difference compared with sample-0 s. Consistent with this, the proliferation-related genes showed the same trend. As shown in Figures 4C–E, the markers of proliferation (PCNA, CDK2, and cyclin A2) were lower expressed in SBS-An films than in normal condition. While p21, acting as an inhibitor of cell cycle progression, was significantly increased in SBS-An films compared with in normal condition(Figure 4B). Together, these results indicate that SBS-An films with different height in the thickness direction have effect on the proliferation of HUVECs.

### Effect of the Aligned Patterned Biomimetic Elastic Membrane on Angiogenesis Related Gene Expression

**FIGURE 5:**
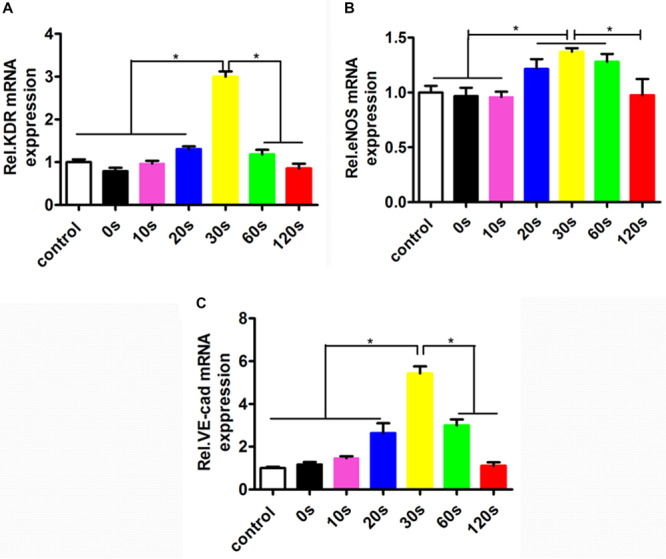
The mRNA expressions of the angiogenesis-related genes in the HUVECs cultured in normal condition and on the sample-0, 10, 20, 30, 60, and 120 s for 72 h. The angiogenesis-related genes expression in HUVECs shown in each panel were **(A)** KDR, **(B)** eNOS, and **(C)** VE-Cad, respectively (*p < 0.05).

To investigate the effect of the aligned patterned biomimetic elastic membrane on angiogenesis related genes, the expression of KDR, eNOS, and VE-Cadherin from HUVECs cultured on biomimetic elastic membrane with different aligned patterns has been investigated. Figure 5 showed the mRNA expression of angiogenesis-related genes(KDR, eNOS and VE-Cad). The mRNA expression of KDR and VE-Cad were siginificantly higher in sample-30 s than in other samples and normal condition (Figures 5A,C). While, the mRNA expression of eNOS in sample-20, 30, and 60 s were significant differences compared with in sample-0, 10,120 s and normal condition. In a way, these results indicate that SBS-An films with different height in the thickness direction have effect on the differentiation of HUVECs.

## Discussion

With the development of micro/nano processing technology, many new methods such as soft lithography, laser particle beam engraving, electron beam etching, and hot stamping have been used to prepare morphologies on the surface of various substrates. There are three types of micro-nano pattern structures currently studied: micro-nano stripe/groove structure, micro-nano bump array, and micro-nano groove array. Among them, the micro-nano level stripe/groove structure has attracted much attention because it can make cells “contact guide” effect (Weiss and Hiscoe, 1948). Lei et al. used photolithography to prepare striped structures with different pitch sizes in polymer templates, and studied the effects of surfaces with striped structures on the adhesion, orientation, and morphological changes of endothelial cells (Lei et al., 2012). However, it is worth mentioning that the aforementioned micro-pattern preparation methods have their own limitations and disadvantages. For example, the requirements for the equipment are relatively high (electron beam etching), and they are limited by the limiting wavelength of the light source (lithography). It is difficult to control the elastic deformation of the mold during operation to ensure the accuracy and repeatability of the pattern replication (soft lithography). In particular, due to the limited number of materials suitable for these technologies, it is not possible to directly achieve the controllable preparation of the micro-patterned structure on the surface of the required biodegradable material. In this work, the patterns were achieved via the UV light irradiation. This method is easy and convenient to operate, and can produce a variety of patterns. Meanwhile, the patterns can be design via the masks as needed.

Studies show that the choice of vascular endothelial cell carrier material and its traits have important effects on cell adhesion, growth, and expression of physiological functions (Jankowski and Wagner, 1999). In order to improve the adhesion and growth of endothelial cells on the surface of artificial blood vessels, people have tried to attach various cell adhesion factors such as collagen, fibronectin, and laminin to the surface of vascular materials, and achieved relatively satisfactory results (Bellis, 2011). Later, it was discovered that the RGD (Arg-Gly-Asp) tripeptide is the smallest sequence for intercellular recognition shared by many cell membranes and multiple adhesion proteins in ECM, which plays a very important role in mediating cell adhesion and spreading (Pierschbacher and Ruoslahti, 1984; Ruoslahti, 1996; Arnaout et al., 2005). Currently, RGD has been widely used in the surface modification of biological materials to promote the adhesion and function of cells on the surface of biological materials (Barber et al., 2007). In addition to attaching various cell activation factors to the surface of the material, it has been found that the necessary 3D structure on the surface of the material is also one of the indispensable factors to promote cell adhesion and growth, especially to maintain cell functional differentiation. Numerous studies have well demonstrated that the appropriate surface topography of scaffolds could promote various cellular processes, including adhesion, proliferation, and migration (Dalby et al., 2007; Kirmizidis and Birch, 2009; Qi et al., 2010; Yang et al., 2010). Tunstall et al. (1994) compared the expression of tissue factor (TF) and prostacyclin (PG) during the growth of vascular endothelial cells on the surface of Dacron materials with different surface structures. During growth, the expression level of TF was significantly lower than that of endothelial cells grown on smooth surface Dacron membranes, while the expression level of PG was not significantly different between the two groups. Sun et al. have already demonstrated that the cell attachment would be greatly influenced by the diameter of electrospun fibers (Sun et al., 2007). Xu et al. (2015) investigated the effect of the fibrous patterns on cells, and found that the well-designed scaffolds with anisotropically and heterogeneously aligned patterns could significantly promote EC adhesion at the early stage and proliferation during the culture period.

Consistent with these studies, our results showed that biomimetic elastic membrane with different height in the thickness direction had an important influence on the adhesion and proliferation of HUVECs, which can significantly improve cell adhesion just inoculated cells, and cell proliferation during the cultivation process. However, the adhesion and proliferation of HUVECs cultured in SBS-An films were lower than these in normal condition. Therefore, future studies will try to attach some cell adhesion factors or the appropriate surface topography on the SBS-An films to improve the adhesion and growth of HUVECs on the biomimetic elastic membrane.

Moreover, we found that the biomimetic elastic membrane had a significant impact on the expression of the proliferation-related genes (such as p21, PCNA, CDK2, and cyclin A2) and angiogenic genes (such as eNOS, KDR, and VE-Cadherin). The tumor suppressor protein p21 Waf1/Cip1 acted as an inhibitor for cell cycle progression, and functioned in stoichiometric relationships to form heterotrimeric complexes with cyclins and cyclin-dependent kinases. In association with CDK2 complexes, it served to inhibit kinase activity and block progression through G1/S (Pestell et al., 1999). Proliferating cell nuclear antigen (PCNA) is a member of the DNA sliding clamp family of proteins that assist in DNA replication (Kelman and O’Donnell, 1995). PCNA expression is a well-accepted marker of proliferation). A number of studies have described the ability of over-expressed cyclin A to accelerate the G1 to S transition causing DNA replication, and cyclin A antisense DNA can prevent DNA replication (D’Urso et al., 1990; Zindy et al., 1992; Resnitzky et al., 1995). eNOS is a key enzyme for endothelial cells to produce NO. The expression level of eNOS can reflect the ability of endothelial cells to secrete NO and is an indicator of endothelial cell integrity and vitality (Sessa, 2004). Upregulation of KDR can promote the survival, proliferation, and differentiation of endothelial cells, and is a better indicator of endothelial cell proliferation and differentiation (Santos et al., 2007). VE-cadherin is expressed at the cell adhesion junction, and its expression can specifically reflect the differentiation of endothelial cells (Schäfer et al., 2003). Examination of the abundance of the above gene expression can prove that biomimetic elastic membrane has an effect on the proliferation and differentiation of HUVECs. Different expression of these genes in SBS-An films indicated that the surface height of the biomimetic elastic membrane may have a significant influence on it (Figures 4, 5). According to the above results, the sample-20,30, and 60 s(height 2.56 ± 0.09, 4.47 ± 0.08, 7.22 ± 0.05 μm, respectively) had a stronger effect on the adhesion and proliferation of HUVECs, in contrast, sample-0,10, and 120 s (0, 1.36 ± 0.12, and 22.86 ± 0.28 μm, respectively.) had no siginificant difference. Therefore, this may be due to that too low or too high of the surface was not good for cell adhesion. However, the exact mechanism needs further study.

In conclusion, we easily achieved well-ordered surface patterned biomimetic elastic membrane with different height in the thickness direction through UV light-induced dimerization of anthracene grafted on SBS chains. All six membranes were found to be non-toxic against HUVECs. Among of them, sample-30 s had siginificantly effect on adhesion and proliferation of the HUVECs, and expression of the proliferation-related and angiogenic genes as compared with other samples, which makes it the best candidate for further improvement. Our results suggest that well-ordered surface-patterned biomimetic elastic membrane might have a potential in vascular tissue-engineering application.

The repair and reconstruction of tissues and organs make tissue engineering a hot research topic. However, the internal tissues and organs of organisms are complex structures with specific morphology and function composed of different cells. Previous methods of experiments are difficult to reconstruct the specific topological conformation of cells in tissues and organs and simulate the microenvironment in which cells are located. After the continuous development of chemical technology, it has gradually become an experimental tool for studying and controlling cell behavior, making it an asset in the fields of cell biology, tissue engineering, cell sensing, drug screening, and wound treatment. Although some defects are still present, such as its non-biodegradability, SBS-An films have many advantages, such as good mechanical properties, low cost, abundance, good biocompatibility, precise and controllable surface patterns, diversified patterns, fast and easy preparation method, and good flexibility, making them a great potential tissue engineering vascular material.

## Data Availability Statement

The original contributions presented in the study are included in the article/supplementary material, further inquiries can be directed to the corresponding author.

## Ethics Statement

The studies involving human participants were reviewed and approved by the Ethics Committees of Shanghai Jiao Tong University. The patients/participants provided their written informed consent to participate in this study.

## Author Contributions

JT and JB carried out the experiments, analyzed the results, and wrote the manuscript, and were co-first authors. All authors contributed to conception and design of the study, and contributed to manuscript revision, read and approved the submitted version.

## Conflict of Interest

The authors declare that the research was conducted in the absence of any commercial or financial relationships that could be construed as a potential conflict of interest.
